# Identification of a novel lactylation-related gene signature predicts the prognosis of multiple myeloma and experiment verification

**DOI:** 10.1038/s41598-024-65937-x

**Published:** 2024-07-02

**Authors:** Cheng Sun, Wanqiu Zhang, Hao Liu, Yangyang Ding, Jingjing Guo, Shudao Xiong, Zhimin Zhai, Wei Hu

**Affiliations:** 1 College of Pharmacy, Anhui Medical University, Hefei, Anhui People’s Republic of China; 2 Department of Clinical Pharmacology, The Second Affiliated Hospital of Anhui Medical University, Hefei, Anhui People’s Republic of China; 3 Department of Hematology/Hematological Lab, The Second Affiliated Hospital of Anhui Medical University, Hefei, Anhui People’s Republic of China

**Keywords:** MM, Lactylation‐related genes, Prognostic signature, PFN1, Cancer, Data mining, Databases

## Abstract

Multiple myeloma (MM) is an incurable hematological malignancy with poor survival. Accumulating evidence reveals that lactylation modification plays a vital role in tumorigenesis. However, research on lactylation-related genes (LRGs) in predicting the prognosis of MM remains limited. Differentially expressed LRGs (DELRGs) between MM and normal samples were investigated from the Gene Expression Omnibus database. Univariate Cox regression and LASSO Cox regression analysis were applied to construct gene signature associated with overall survival. The signature was validated in two external datasets. A nomogram was further constructed and evaluated. Additionally, Enrichment analysis, immune analysis, and drug chemosensitivity analysis between the two groups were investigated. qPCR and immunofluorescence staining were performed to validate the expression and localization of PFN1. CCK-8 and flow cytometry were performed to validate biological function. A total of 9 LRGs (TRIM28, PPIA, SOD1, RRP1B, IARS2, RB1, PFN1, PRCC, and FABP5) were selected to establish the prognostic signature. Kaplan–Meier survival curves showed that high-risk group patients had a remarkably worse prognosis in the training and validation cohorts. A nomogram was constructed based on LRGs signature and clinical characteristics, and showed excellent predictive power by calibration curve and C-index. Moreover, biological pathways, immunologic status, as well as sensitivity to chemotherapy drugs were different between high- and low-risk groups. Additionally, the hub gene PFN1 is highly expressed in MM, knocking down PFN1 induces cell cycle arrest, suppresses cell proliferation and promotes cell apoptosis. In conclusion, our study revealed that LRGs signature is a promising biomarker for MM that can effectively early distinguish high-risk patients and predict prognosis.

## Introduction

Multiple myeloma (MM) is a hematological malignancy characterized by the abnormal expansion and accumulation of monoclonal plasma cells in the bone marrow and the production of large amounts of abnormal monoclonal immunoglobulins^1^. It accounts for approximately 10% of all hematological malignancies and mainly occurs in the elderly, and is still incurable. Over the past ten years, with the emergence of new drugs and innovations in chemotherapy regimens, MM patient’s prognosis has greatly improved^2,3^. Due to the obvious heterogeneity in the pathogenesis and clinical manifestations of MM, part of patients may still experience disease recurrence and exacerbation^4^. The current International Staging System (ISS) is based albumin and β2-microglobulin, and is widely used in clinical practice^5^. However, in the management of MM patients, this system still cannot accurately guide patients' individualized treatment and prognosis. In view of the limitations of the current staging system, it is necessary to identify novel biomarkers and establish a prognostic model based on ISS to predict outcomes and help select appropriate treatment regimens.

Protein lactylation is a novel identified post-translational modification, first reported in 2019, which is considered to be an important way of linking metabolism to gene expression^6^. Numerous studies have shown that lactylation modification plays a vital role in biological processes such as inflammation, immune regulation, embryogenesis, and oncogenic processes^7^. In addition, high levels of protein lactylation are associated with poorer prognosis in ocular melanoma and clear cell renal cell carcinoma^8,9^. Mechanistically, it is found that lactylation modification participates in tumorigenesis by regulating immunity, metabolic reprogramming and other ways^6,10,11^. Especially in the tumor microenvironment, the tumor metabolite lactate is a key factor driving and controlling the level of protein lactylation and affects gene expression, and lactylation modification may be a crucial factor in the growth of tumors^12^. In MM, there has been numerous studies on the role of lactate in malignant cell proliferation and drug resistance, and lactate promotes protein lactylation, which is a key factor in promoting resistance^13,14^. A study has shown that exogenously increased lactate promotes insulin like growth factor receptor-1 (IGF-1R) lactylation, while repressing IGF-1R lactylation inhibits MET proto-oncogene, receptor tyrosine kinase (MET) activation and weakens the proteasome inhibitors resistance of MM cells^15^. These mechanisms suggest that targeting lactylation and related pathways, may be a potential strategy for treating MM. However, the role of lactylation-related genes (LRG) has not been fully elucidated, and its relationship with the prognosis of MM patients remains unclear.

In this study, we extracted the expression profiles of LRGs in MM and further constructed a LRGs prognostic signature associated with overall survival (OS) by univariate Cox and LASSO regression analysis in the training cohort, and validated in two external cohorts. We further constructed a nomogram based on patient age, ISS, and LRGs signature, and evaluated the performance by calibration curve and C-index. We predicted the response to chemotherapeutic drugs and comparative analyses of biological pathway and tumor microenvironment (TME) to explore the underlying mechanisms in different risk MM patients. In addition, the expression, localization and biological function of PFN1 have been further explored. This is the first study to construct a useful and feasible LRGs signature, which can be used to predict MM outcomes and early distinguish high-risk patients.

## Materials and methods

### Data source and screening of DELRGs

MM-related gene expression data and clinical information were downloaded from the Gene Expression Omnibus (GEO) (https://www.ncbi.nlm.nih.gov/geo/) database. The GSE6477 dataset, which contains 15 normal and 69 newly diagnosed MM patients, was used to identify DEGs. The GSE2658 dataset, consisting of 559 MM patients, was utilized as the training cohort. Additionally, the GSE136337 and GSE9782 datasets included 426- and 264-MM patients respectively and were used as external validation cohorts. The clinical features of MM patients from different cohorts are summarized in Table 1. The lactylation-related genes were gathered from previously published studies^6,16,17^. After deleting duplicate genes, we identified a total gene set of 332 genes and are presented in Supplementary Table S1. All transcriptomic data were standardized and normalized, and DEGs were analyzed by “limma” package (version 3.5.1). The DEGs threshold was set as follows: |logFC)|> 0.585 and an adjusted *P*-value < 0.05. Volcano Plot and heatmap were conducted with the “ggplot2” and “pheatmap” packages for visualizing the DEGs.

**Table 1:** Clinical parameter characteristics of the MM patients retrieved from the GEO databases.

Characteristics		GSE2658	GSE136337	GSE9782
Category		Training cohort	Validation cohort	Validation cohort
Platform		GPL570	GPL27143	GPL96
Total number		559	426	264
Survival status	Survival	459 (82.1%)	243 (57.0%)	109 (41.3%)
	Death	100 (17.9%)	183 (43.0%)	155 (58.7%)
Age	> 65	126 (22.5%)	119 (27.9%)	80 (30.3%)
	≤ 65	433 (77.5%)	307 (72.1%)	184 (69.7%)
Sex	Male	336 (60.1%)	261 (61.3%)	159 (60.2%)
	Female	223 (39.9%)	165 (38.7%)	105 (39.8%)
ISS	I	318 (56.9%)	170 (39.9%)	69 (26.1%)
	II	121 (21.6%)	135 (31.7%)	64 (24.2%)
	III	120 (21.5%)	121 (28.4%)	61 (23.1%)
	Unknow	–	–	70 (26.5%)

Annotation: GPL27143, [HG-U133_Plus_2] Affymetrix Human Genome U133 Plus 2.0 Array; GPL96, [HG-U133A] Affymetrix Human Genome U133A Array; GPL570, [HG-U133_Plus_2] Affymetrix Human Genome U133 Plus 2.0 Array.

### Functional annotation analysis

Gene set enrichment analysis (GSEA) was analyzed by the ‘clusterProfiler’ package. The annotated gene sets of GSEA were selected, c2.cp.kegg.v2023.1.Hs.entrez and c5.go.bp.v2023.1.Hs.entrez sets from the Molecular Signature Database (MSigDB) (https://www.gsea-msigdb.org/gsea/msigdb/). The number of permutations was set to 1000. The criteria for screening statistically significant pathways were set as *P*-value < 0.05.

### Gene signature construction and validation

LASSO regression analyses were used to screen out DELRGs involved in the prediction signature in training cohort, *P*-value < 0.05 was considered statistically significant. The least absolute shrinkage and selection operator was used to construct the DELRGs signature, and the risk score model trained from the GEO data was constructed as follows: $\text{Riskscore}={\sum }_{i=1}^{N}(\text{exp}\times coef),$ where *N* is the number of model genes; exp represents the gene expression value of each gene; *coef* represent the coefficient index. Kaplan–Meier survival curves and time-dependent receptor operating characteristic (ROC) curve were plotted to evaluate the predictive power of the prognostic signature. The “rms” package to construct a nomogram containing each LRGs, which was further evaluated the performance by calibration curve.

### Immune infiltration assessment

The “GSVA” package was used to perform single sample gene set enrichment analysis (ssGSEA) algorithm to calculate the infiltration score of tumor-infiltrating immune cells and functions in high- and low-risk patients, and visualized the results using the “vioplot” package.

### Treatment responsiveness evaluation

To predict chemosensitivity between high-risk and low-risk groups, we utilized the “pRRophetic” package (version 0.5), primarily mainly construct ridge regression model to infer half-maximal inhibitory concentration (IC50) values based on gene expression levels through ten-fold cross-validation^18,19^. The dataset within the "pRRophetic" package is derived from the “cgp2016” initiative, encompassing gene expression matrices and drug treatment information, including 251 drugs and 13 myeloma cell lines. We analyzed common anticancer drugs (doxorubicin, bortezomib and navitoclax) based on clinical experience. The difference in drug sensitivity between low-risk and high-risk groups was illustrated using box plots.

### Patient samples and cell culture

20 newly diagnosed MM, 20 complete remission MM and 7 healthy control bone marrow samples were obtained from the Second Affiliated Hospital of Anhui Medical University. MM patient’s clinical characteristics were summarized in Supplementary Table S2. The study was approved by the Ethics Committee of the Second Hospital of Anhui Medical University, and informed consent was obtained from the patients. All experiments were performed in accordance with relevant guidelines and regulations. Human MM cell lines H929 was purchased from the Institute of Biochemistry and Cell Biology of the Chinese Academy of Science. Cells were cultured in RPMI-1640 (Hyclone, Logan, UT, USA) containing 10% FBS at 37 °C cell incubators with 5% CO_2_.

### SiRNA transfection

MM cells were transfected with PFN1 siRNA and negative control siRNA (GenePharma, China) using LipofectamineTM 3000 (Invitrogen, USA) reagent following the manufacturer's instructions. Briefly, 4 × 10^5^ cells were seeded in a 24-well plate, dilute 4 µL siRNA (20 uM) with 50 µL Opti-MEM, and dilute 1 uL LipofectamineTM 3000 with 50 uL Opti-MEM. Mix the transfection reagent and siRNA diluent and add 24-well plate, continue to culture for 6 h, change the medium, and after 24 h of transfection, expression of PFN1 was verified by subsequent assay. PFN1 siRNA sequence was as follows: sense 5ʹ-AGA AGG UGU CCA CGG UGG UUU-3ʹ; antisense 5ʹ-ACC ACC GUG GAC ACC UUC UUU -3’. Negative control siRNA sequence was as follows: sense 5ʹ-UUC UCC GAA CGU GUC ACG UTT-3ʹ; antisense 5ʹ-ACG UGA CAC GUU CGG AGA ATT-3ʹ.

### Quantitative PCR

Total RNA was extracted using Trizol reagent (sangon Biotech, Shanghai) according to the manufacturer’s protocol, and cDNA was synthesized using RevertAid First Strand cDNA Synthesis Kit (Thermofisher, USA). qPCR was conducted in ABI 7500 System (Life Technologies, USA) using SYBR Green Mix. The reaction conditions are as follows: predenaturation: 1 cycle of 95 °C for 30 s, amplification: 40 cycles of 95 °C for 5 s and 60 °C for 30 s, melting curve: 1 cycle of 95 °C for 15 s and 1 cycle of 60 °C for 60 s. cooling: 1 cycle of 50 °C for 30 s. The relative expression was determined using the 2^−ΔΔCt^ method, with GAPDH as an endogenous control. Primers for PFN1 mRNA were as follows: forward 5ʹ-GTTCGTCAACATCACGCCAG-3ʹ; reverse: 5ʹ-GTCCCGGATCACCGAACATT-3ʹ. Primers for GAPDH mRNA: forward 5ʹ-AGCAAGAGCACAAGAGGAAG-3ʹ; reverse: 5ʹ-GGTTGAGCACAGGGTACTTT-3ʹ.

### Western blot

Whole cell lysates were prepared using RIPA buffer. Protein concentration was quantified using Enhanced BCA Protein Assay Kit (Beyotime biotechnology). Equal amounts of protein were blotted and separated by SDS-PAGE and transferred to NC membranes, and after blocking the membranes with 5% skim milk, the membranes were incubated overnight with one of the antibodies listed in Supplementary Table S3. Then incubate with corresponding HRP-conjugated secondary antibodies. Immunoreactive bands were detected with WesternBright ECL kit (Advansta, USA). Grayscale analysis of WB bands was performed using ImageJ software (v.1.50a) (https://imagej.net/software/imagej/).

### Cell viability assay

Cells were cultured for 24 h, 48 h, 72 h with complete medium, and cell viability was measured using CCK8 reagent (Beyotime, China) according to the manufacturer’s protocol. Absorbance was measured at 450 nm using multiscan spectroscopy.

### Cell apoptosis and cell cycle assay

According to the manufacturer’s protocol, cell apoptosis was detected by Annexin V-FITC/PI Apoptosis Detection Kit (BestBio, China), and cell cycle was detected by Cell Cycle Staining Kit (Lianke Biotech, China). The fluorescence of at least 5,000 cells per sample was measured on a Cytomics FC 500 flow cytometer (Beckman Coulter, USA) for further calculations.

### Immunofluorescence staining

Mononuclear cells were extracted from the bone marrow, fixed on the slide, permeabilized with 0.1%TritonX-100 for 15 min, and then blocked with 10% goat serum in tris-buffered saline (TBS) for 30 min at room temperature. The sections were incubated overnight at 4 °C with the rabbit anti-PFN1 primary antibody (1:200, Proteintech, China), and then incubated with the secondary antibody FITC goat anti-rabbit IgG (1:200; Abcam, USA) for 1 h. After PBST wash 3 times, APC-CD38 (1:50, Beckman Coulter Life Sciences, USA) were incubated for 30 min. The sections were counterstained with DAPI (1:100; Beyotime, China). The slides were visualized with a confocal microscopy (Olympus FV10i, Tokyo, Japan).

### Statistical analysis

All statistical analyses were performed using the R software (v.4.2.1). Student’s t-test were used to compare the differences between the two groups. Survival curves were generated by the Kaplan–Meier method and compared using the log-rank test. Multivariate Cox proportional hazards regression was used to identify independent prognostic factors. The results were presented as the mean ± standard deviation (SD) of at least three independent experiments.* P* < 0.05 was considered statistically significant.

### Ethics approval and consent to participate

This research was approved by the institutional ethics Committee of The Second Affiliated Hospital of Anhui Medical University, informed consent was obtained from all patients.

## Results

### Establishment of LRGs prognostic signature

**Figure 1:**
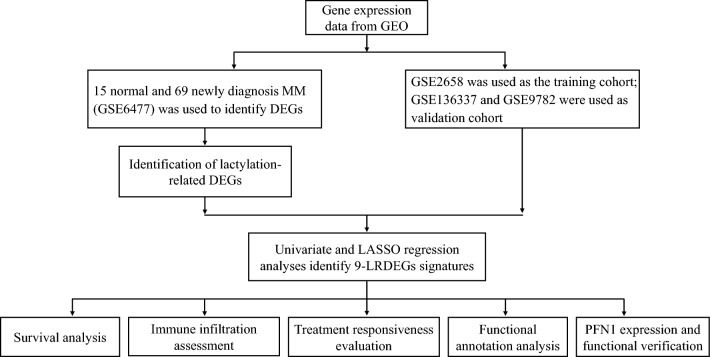
Study flow chart.

**Figure 2:**
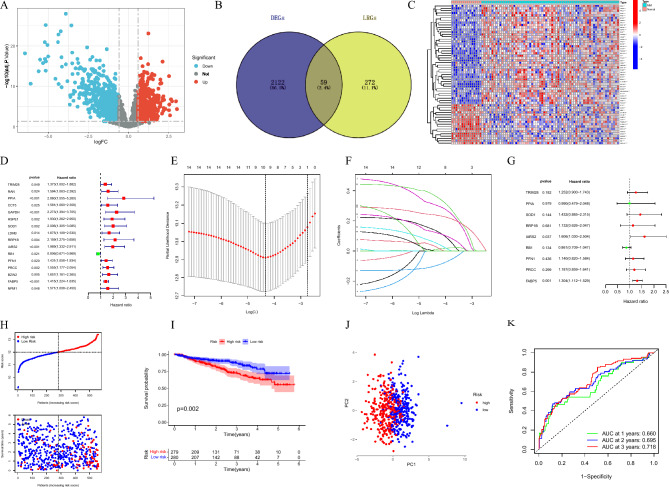
Establishment of LRGs prognostic signature. (**A**) Volcano plot of DEGs; (**B**,**C**) Venn diagram and heatmap of DELRGs; (**D**) Forest plot of univariate Cox regression analysis in the training cohort; (**E**,**F**) LASSO regression analysis for most suitable λ; (**G**) Forest plot of multivariate Cox regression analysis; (**H**) The distribution of risk scores and survival status of MM in the training cohort; (**I**) Kaplan–Meier survival curve of high- and low-risk in the training cohort; (**J**) PCA plot in the training cohort; The red dots represent high-risk patients and blue dots represent low-risk patients; (**K**) ROC curve of 1, 2, 3-years survival prediction in the training cohort.

The flow chart of the study is shown in Fig. 1. To establish the prognostic signature of LRGs, we set a relaxed threshold |logFC|> 0.585 and an adjusted *P-*value < 0.05, and a total of 59 DELRGs were identified based on the gene expression set of 15 normal and 69 newly diagnosed MM bone marrow samples in the GSE6477 dataset, as shown in Supplementary Table S4 and Venn diagram (Fig. 2A,B). The profiles of DELRGs were displayed in heatmap plots (Fig. 2C). We utilized GSE2658 datasets as training cohort and investigated the association between 59 DELRGs and MM prognosis. Univariate Cox regression results showed that 16 genes were associated with MM prognosis. We further conducted multivariate Cox regression and LASSO regression to construct a prognostic signature (Fig. 2D–G). When the model reached the minimum of lambda (λ), an optimal gene prognostic signature, with 9 non-zero coefficient genes (TRIM28, PPIA, SOD1, RRP1B, IARS2, RB1, PFN1, PRCC, and FABP5) was identified. The LRGs signature risk score was as follows: Risk score = (0.1318 × expression level of TRIM28) + (0.0114 × expression level of PPIA) + (0.3025 × expression level of SOD1) + (0.0247 × expression level of RRP1B) + (0.3596 × expression level of IARS2) + (− 0.0960 × expression level of RB1) + (0.0505 × expression level of PFN1) + (0.1209 × expression level of PRCC) + (0.2374 × expression level of FABP5). Based on the calculated risk score of each patient, patients were divided into high- and low-risk groups. As shown in Fig. 2H, more deaths were observed in MM patients with high-risk group. Kaplan–Meier survival analysis showed that high-risk group patients exhibited a worse OS compared to low-risk group patients in the training cohort (HR 3.603, 95% CI 2.468–5.261, *P* < 0.001, Fig. 2I). PCA analysis results showed that the LRGs signature scores was able to accurately distinguish high-risk patients (Fig. 2J). Next, for OS prediction, the AUC at the 1-, 2-, 3-years ROC curve was 0.660, 0.695, 0.718, respectively, which demonstrated that the LRGs signature exhibited high predictive power in the training cohort (Fig. 2K).

### Validation of LRGs prognostic signature

**Figure 3:**
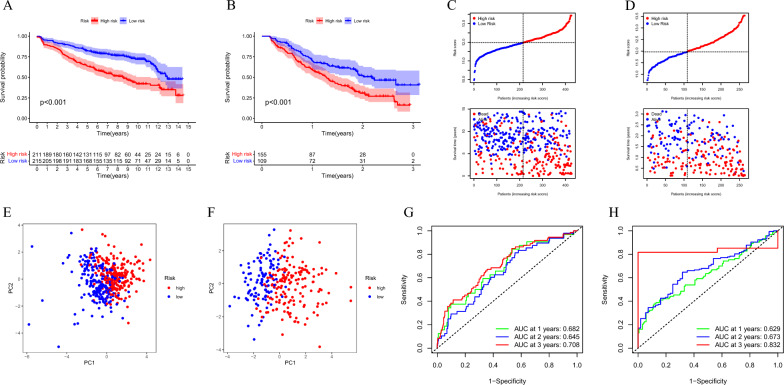
Validation of LRGs prognostic signature. (**A**,**B**) Kaplan–Meier survival curve of high- and low-risk in the GSE136337 and GSE9782 validation cohort; (**C**,**D**) The distribution of risk scores and survival status of MM in the validation cohort; (**E**,**F**) PCA plot in the validation cohort; The red dots represent high-risk patients and blue dots represent low-risk patients; (**G**,**H**) ROC curve of 1, 2, and 3-years survival prediction in the validation cohort.

**Figure 4:**
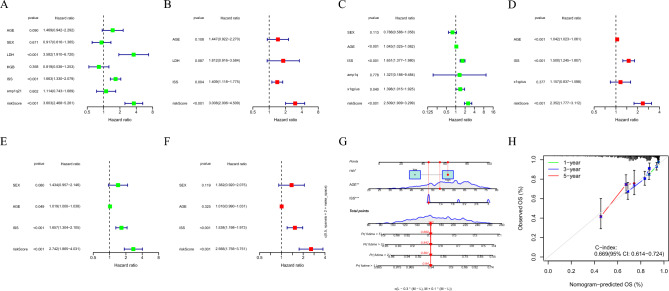
Forest plots of univariate and multivariate Cox regression analyses, nomograms and calibration curves based on clinical parameters and risk score. (**A**,**B**) Forest plot of the univariate and multivariate Cox regression analysis of the clinical parameters and risks core in the GSE2658 training cohort; (**C**,**D**) Forest plot of the univariate and multivariate Cox regression analysis of the clinical parameters and risks core in the GSE136337 validation cohort; (**E**,**F**) Forest plot of the univariate and multivariate Cox regression analysis of the clinical parameters and risks core in the GSE9782 validation cohort; (**G**) Nomogram based on clinical parameters and LRGs signature; The total score of the 3 variables is projected on the bottom scale to represent the probability of overall survival at 1-, 2-, 3-, and 5-years; (**H**) Calibration curve of 1-, 3-, and 5-years OS.

To further validate the robustness of the LRGs signature, we utilized GSE136337 and GSE9782 datasets as validation cohorts, and consistently found that the high-risk group patients, stratified using the same calculation formula as the training cohort, was significantly associated with worse overall survival (OS) in both the GSE136337 validation cohort (HR 2.509, 95% CI 1.909–3.299, *P* < 0.001; Fig. 3A) and the GSE9782 validation cohort (HR 2.742, 95% CI 1.865–4.031, *P* < 0.001; Fig. 3B), and more deaths were observed in MM patients with high-risk group in the validation cohort too (Fig. 3C,D). The LRGs signature score was able to accurately distinguish high-risk patients (Fig. 3E,F). In addition, for OS prediction, in the GSE136337 validation cohort, the AUC at the 1-, 2-, 3-years ROC curve was 0.682, 0.645, 0.708 (Fig. 3G). In the GSE8782 validation cohort, the AUC at the 1-, 2-, 3-years ROC curve was 0.629, 0.673, 0.832 (Fig. 3H). These results indicate that LRGs signature have excellent predictive value. In addition, based on the univariate and multivariate analysis results showed that risk score and ISS are independent prognostic factors in the training and validation cohorts (Fig. 4A–F), and we constructed a nomogram based on patient age, ISS, and LRGs signature (Fig. 4G). The calibration curve verified the accuracy of the LRGs signature for predicting 1-, 3-, 5-years survival rates (Fig. 4H), and proved that the value of the nomogram was a good predictive tool for MM prognosis.

### Sensitivity to chemotherapy in high- and low-risk patients

**Figure 5:**
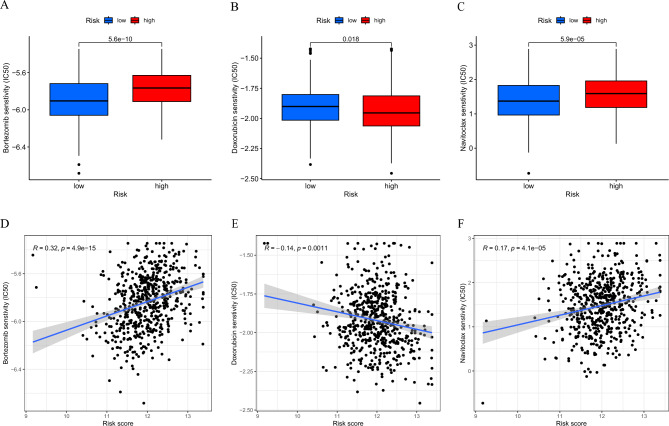
Sensitivity to chemotherapy in high- and low-risk patients. (**A**–**C**). Sensitivity to conventional chemotherapy drugs (bortezomib, doxorubicin, and navitoclax) for MM patients in high- and low-risk patients; (**D**–**F**) Correlation analysis between risk scores and IC50 of conventional chemotherapy drugs.

To investigate the sensitivity of high- and low-risk patients to chemotherapeutic agents, we predicted the IC50 of 130 chemotherapy drugs and pathway inhibitors based on the pRRophetic algorithm and found that the IC50 of conventional chemotherapy drugs doxorubicin was lower in high-risk patients, which indicated that high-risk patients are more sensitive to doxorubicin. The IC50 of bortezomib and navitoclax were lower in low-risk patients, suggesting that low-risk patients may benefit from these drugs (Fig. 5A–F). The above results suggest that the LRGs signature may help patients select appropriate chemotherapy drugs.

### Functional enrichment analysis and immune infiltration analysis

**Figure 6:**
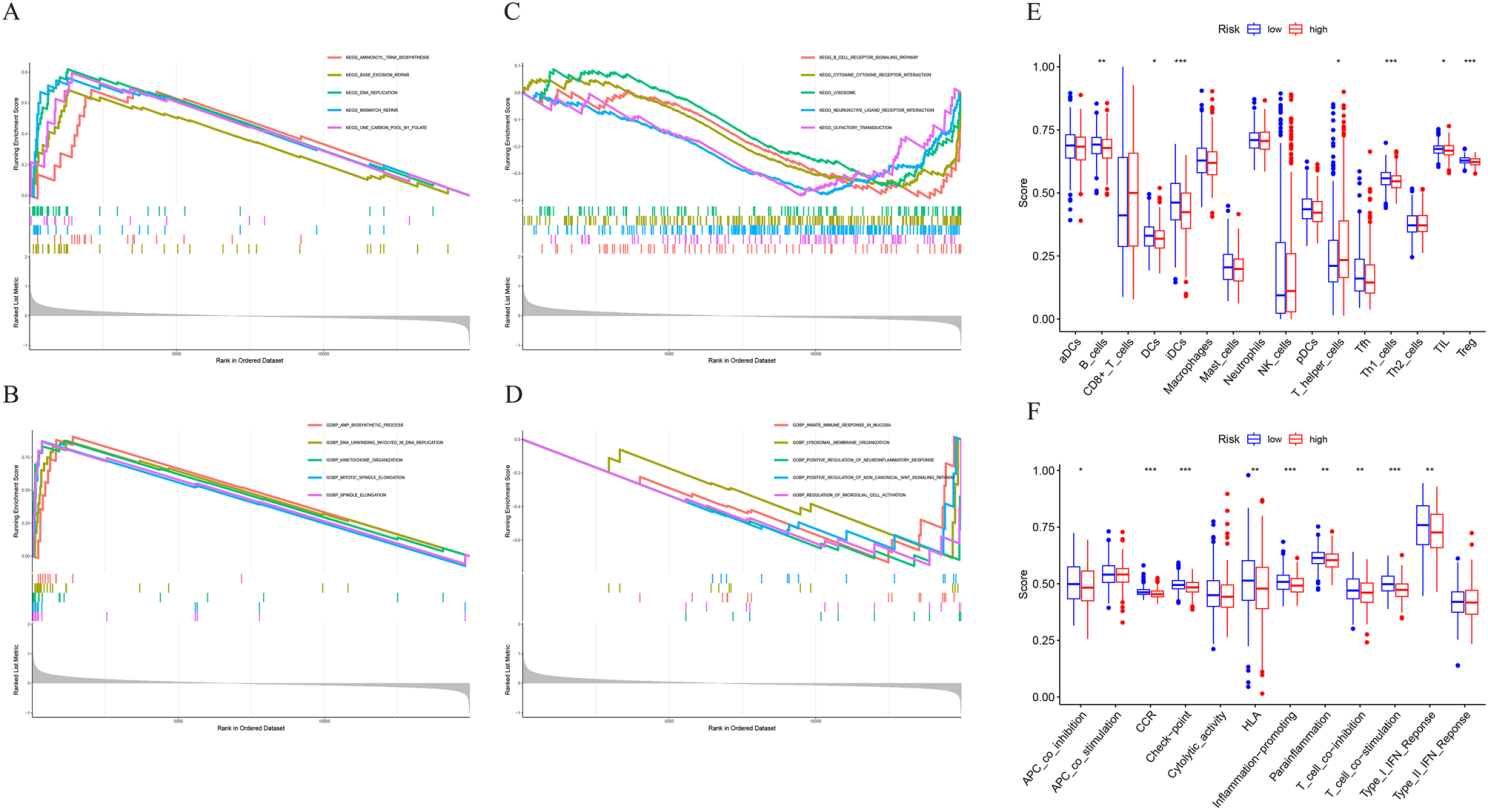
Functional enrichment analysis and immune infiltration analysis. (**A**,**B**) GSEA KEGG analysis in high- and low-risk patients; (**C**,**D**) GSEA GO analysis in high- and low-risk patients; (**E**,**F**) Difference of infiltrating immune cell types and functions in high- and low-risk patients.

GSEA was conducted to investigate the potential biological pathways between two risk groups. The results showed that positive regulation of non-canonical Wnt signaling pathway, B-cell receptor signaling pathway, and cytokine-cytokine receptor interaction were significantly enriched in the low-risk group (Fig. 6A,B). While biological processes such as AMP biosynthetic process, DNA unwinding involved in DNA replication and signaling pathways such as aminoacyl tRNA biosynthesis and DNA replication were significantly enriched in the high-risk group (Fig. 6C,D). Numerous studies have shown that TME is closely related to tumor progression and immunotherapy response. TME is mainly reflected in the types and functions of immune cells in tumor tissues. To further understand the differences in immune cell abundance between two risk groups, we used ssGSEA algorithm to explore the TME in MM. The analysis revealed that 7 immune cell types, including B cells, DCs, iDCs, T helper cells, Th1 cells, TIL, and Treg, and 9 immune pathways, including APC co-inhibition, CCR, check-point, HLA, inflammation-promoting, parainflammation, T cell co-inhibition, T cell co-stimulation, Type I IFN response, were significantly different in high-risk and low-risk groups (Fig. 6E,F). The results suggest that the LRGs signature reflects the immune status of the TME in different risk group MM patients.

### PFN1 is highly expressed in MM, knocking down PFN1 induces cell cycle arrest, suppresses cell proliferation and promotes cell apoptosis

**Figure 7:**
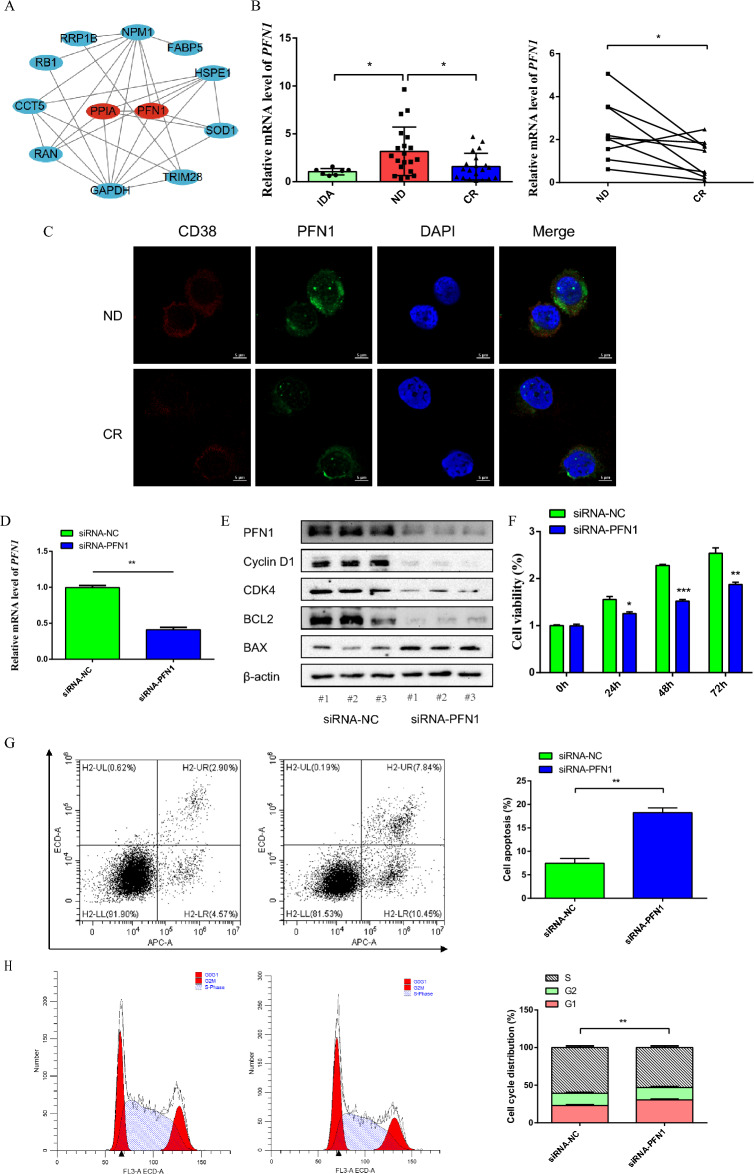
PFN1 is highly expressed in MM, knocking down PFN1 induces cell cycle arrest, suppresses cell proliferation and promotes cell apoptosis. (**A**) Protein‐protein interaction network constructed with DELRGs related to MM prognosis. (**B**) The expression of PFN1 mRNA was measured by qPCR. (**C**) The expression of CD38 + cell PFN1 in the bone marrow of newly diagnosed and completely remission myeloma patients was detected by immunofluorescence. (**D**) qPCR verify PFN1 knockdown efficiency. (**E**) Western blot detect PFN1, CyclinD1, CDK4, BCL2, and BAX protein levels (original blots are presented in Supplementary Fig. 1). (**F**) CCK-8 assay to detect cell viability. (**G**) Flow cytometry to detect cell apoptosis. H. Flow cytometry to detect cell cycle. Data are expressed as mean ± SD (*P < 0.05, **P < 0.01, ***P < 0.001).

To explore the potential functions of LRGs signature genes in MM, we used the STRING database to generate a PPI network for identifying the hub gene, and the results showed that PPIA and PFN1 were hub genes (Fig. 7A). We verified the expression level of PFN1 in MM. qPCR results showed that PFN1 mRNA levels were highly expressed in newly diagnosed MM compared to healthy control (Fig. 7B). Additionally, the expression of PFN1 protein and mRNA levels in newly diagnosed MM were higher than in complete remission MM (Fig. 7B,C). Subsequently, we used commercial siRNA to knock down PFN1 (Fig. 7D). Knocking down PFN1significantly decreased the protein expression of CyclinD1, CDK4, and BCL2, and increased protein expression of BAX in H929 cells. Functionally, knocking down PFN1 suppressed cell proliferation, promoted cell apoptosis, and induced cell cycle arrest in the G1/G0 phase. (Fig. 7E–H). These results indicated that PFN1 plays an important role in the development of MM.

## Discussion

MM is a malignant tumor caused by abnormal expansion of plasma cells. Many studies have noted that lactylation modification plays a crucial role in tumor occurrence and development^10,20^. Research has demonstrated that protein lactylation levels influence various cancer characteristics and LRGs could serve as a reliable indicator of prognosis^16,21^. However, research on LRGs in MM is relatively limited, and the prognostic value of LRGs in MM has not yet been elucidated. Here, we studied LRGs and their potential biological functions involved, further developed a LRGs prognostic signature that can effectively early distinguish high-risk patients and predict clinical outcomes.

In present study, we identified 59 DELRGs based on transcriptomic data from normal and newly diagnosed MM bone marrow sample. Univariate Cox regression analysis results showed that 16 DELRGs were related to the OS, we further established a LRGs prognostic signature (TRIM28, PPIA, SOD1, RRP1B, IARS2, RB1, PFN1, PRCC, and FABP5) in the GSE2658 training cohort. Based on the LRGs signature score, high-risk group MM patients had a worse prognosis than low-risk group patients, and the ROC curve showed that the LRGs signature has a good predictive performance. We also evaluated the predictive performance in multiple external validation cohorts, which showed excellent robustness and reliability. Additionally, the risk score and ISS are independent prognostic factors in the training and validation cohorts. We further constructed a nomogram based on patient age, ISS, and LRGs signature for predicting 1-, 3-, and 5-years OS of MM. Calibration curve analysis and C-index showed that the nomogram was a good predictive tool for MM prognosis. In addition, we analyzed the differences in biological pathways, sensitivity to chemotherapy drugs, and immune infiltration among different risk groups. We found differences in the types and functions of infiltrating immune cells, biological pathways between high- and low-risk groups, as well as sensitivities to chemotherapy drugs such as doxorubicin, bortezomib, and navitoclax.

The 9 gene signature (TRIM28, PPIA, SOD1, RRP1B, IARS2, RB1, PFN1, PRCC, and FABP5) may play an important role in MM. PPIA, SOD1, PFN1, RB1 and FABP5 have been reported in MM, and some genes are related to drug resistance and prognosis. PPIA is the central enzyme in the protein folding reaction pathway and is considered a potential new target for drug-resistant MM. Studies have shown that PPIA is highly expressed in MM and is associated with poor prognosis, inhibition of PPIA significantly sensitizes MM cells to proteasome inhibitors^22^. Furthermore, PPIA is also upregulated in other solid tumors such as bladder, esophageal, renal, hepatocellular, and lung cancer and is associated with poor prognosis^23^. SOD1 is an oxidative stress-inducible gene that mediates redox reactions and regulates bortezomib resistance in MM^24^, and in vitro functional studies have shown that forced expression of SOD1 induces resistance to bortezomib, while knockdown of SOD1 increases the sensitivity of drug-resistant MM cell lines to bortezomib^25^. SOD1 is upregulated in MM and associated with disease progression and shorter survival. It is also linked to the progression and adverse overall outcomes in other cancers^24,26^. PFN1 is considered a key regulator of intracellular actin polymerization. In this study, we verified the expression and biological function of PFN1, and the results showed that PFN1 was abnormally highly expressed in MM, and the expression of PFN1 decreased after MM patients achieved complete remission. This result is consistent with Lu's study. In addition, Lu's study further showed that high expression of PFN1 is correlated with poor prognosis of MM patients. In vitro, forced expression of PFN1 promoted RPMI8226 cell proliferation and resistance to bortezomib by activating the autophagy process, while knockdown of PFN1 blocks autophagy and sensitizes RPMI8226 cell to bortezomib^27,28^. Our results showed that knockdown of PFN1 suppresses H929 cell proliferation, promotes cell apoptosis, and induces cell cycle arrest in the G1/G0 phase. These data indicated that PFN1 plays an important role in the development of MM. Although we have preliminarily explored the expression and biological functions of PFN1, the mechanism of PFN1 involved lactylation in MM pathogenesis and drug resistance urgently needs further exploration. RB1 is considered to be a key protein in regulating the cell cycle, studies have found that homozygous deletion and biallelic inactivation of the RB1 are more common in relapsed MM, and identifying RB1 as an independent poor prognostic marker^29^. FABP5 mainly regulates fat metabolism and transfer, studies have found that FABP5 is an important mediator in increasing resistance to chemotherapy drugs, and high levels of FABP5 correlate with MM aggressiveness and poor prognosis^30^. In other solid tumors, FABP5 promotes the malignant proliferation and metastasis of tumor, such as prostate cancer, breast, cancer, and liver cancer, and are associated with prognosis^31^.

Although little is known about the roles of TRIM28, RRP1B, IARS2, and PRCC in MM, their expression levels are upregulated in high-risk MM patients. TRIM28 is regarded to be involved in the regulation of chromatin structure^32^. Studies have found that TRIM28 abnormally expressed in gastric cancer, colorectal cancer, and non-small cell lung cancer, promotes tumor progression^33–35^. IARS2 mainly catalyzes tRNA aminoacylation and correlates with malignant proliferation and apoptosis resistance of tumor cells. In non-small cell lung cancer, gastric cancer, melanoma and acute myeloid leukemia, knockout of IARS2 inhibits cell proliferation and promotes apoptosis^36–39^. PRCC is an important element involved in the formation of the spliceosome^40^. Studies have found that PRCC is highly expressed in HCC, which makes cancer cells insensitive to DNA damage and increases heterogeneity, and is significantly related to the poor prognosis of HCC patients^41^; RRP1B is regarded as a new candidate susceptibility gene for breast cancer progression and metastasis^42^. Although the role of these genes in solid tumors has been reported, relevant research in MM needs to be further carried out, especially involving lactate metabolism and lactylation modification, which may deepen our understanding of the pathogenesis of MM.

The LRGs signature based on the 9-gene score showed good prognostic prediction performance. To further elucidate the role of LRGs in survival stratification and association with survival in MM patients. GSEA functional enrichment analysis was performed for all genes in the high-risk and low-risk groups. The results revealed that non-canonical Wnt signaling pathway was significantly enriched in the low-risk groups, and aminoacyl tRNA biosynthesis was significantly enriched in the high-risk groups. The non-canonical Wnt signaling is critical for the osteogenic differentiation^43,44^, a study pointed out that activation of the non-canonical Wnt signaling pathway in human mesenchymal stromal cells increases osteogenic differentiation and counteract the osteoclastic effects of MM cells, and further suggest that non-canonical Wnt signaling may be a therapeutic target for MM-related bone disease^45^. An increasing number of studies have shown that tRNA abundance increases significantly in cancer, suggesting that it may be a factor in cancer progression^46^. A study reported that abnormally high translation activity exists in MM cells and is mediated by high abundance of tRNA, and that application of proteasome inhibitors reduced aminoacyl tRNA biosynthesis^47^. In addition, acting on certain tRNA synthetases may serve as new therapeutic targets for MM^48^. The above studies and our results suggest that acting on these biological pathways and processes may ultimately affect the disease progression and the prognosis of MM.

The ISS is a commonly employed MM staging system in clinical practice, providing a more precise evaluation of the prognosis of patients^5^. However, in today's emphasis on individualized and precise assessment of patient prognosis, ISS still has some drawbacks. Our results showed that ISS and risk score are independent prognostic factors, and we further constructed a nomogram based on patient age, ISS, and LRGs signature, which makes predicting patient prognosis more individualized and precise, and is an effective complement to ISS assessment of prognosis. In addition, this nomogram has obvious advantages. First, reproducibility across multiple external datasets. Second, compared with previous studies based on pure gene nomogram to predict the clinical outcomes of MM patients, it combines clinical parameters and LRGs signature, making it both broadly applicable and individually precise^49,50^.

However, numerous limitations in our study that should be considered. First, the LRGs signature was established based on the transcriptome and clinical data of the Caucasians, which may be biased, and the predictive efficacy among different races is unclear and requires further verification. Second, when the LRGs signature is extended to MM patients to participate in clinical trials or new drug treatments, caution needs to be exercised in predicting conclusions. Third, we preliminarily explored the expression and biological functions of PFN1, but the mechanism of PFN1 involved lactylation in the pathogenesis of MM still needs further exploration, and other LRGs also need further verification. Meanwhile, the prognostic value of the signature needs to be further clinically verified.

## Conclusion

To sum up, it was the first study to establish prognostic indicators based on LRGs in MM. The LRGs signature can early distinguish high-risk patients and predict clinical outcomes in multiple independent cohorts. The LRGs signature may be practical and reliable prognostic tools for MM and may help guide the development of alternative therapies.

### Supplementary Information

Supplementary Figure S1.

Supplementary Table S1.

Supplementary Table S2.

Supplementary Table S3.

Supplementary Table S4.

## Data Availability

The datasets used or analyzed during the current study are available from the corresponding author on reasonable request.
