# Supplementary figures and images for: Identification of a novel lactylation-related gene signature predicts the prognosis of multiple myeloma and experiment verification
Source: Sci Rep. 2024 Jul 2;14:15142. doi: 10.1038/s41598-024-65937-x (PMC11219856; doi:10.1038/s41598-024-65937-x)

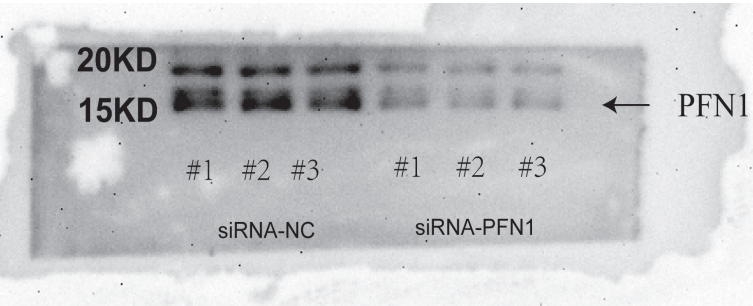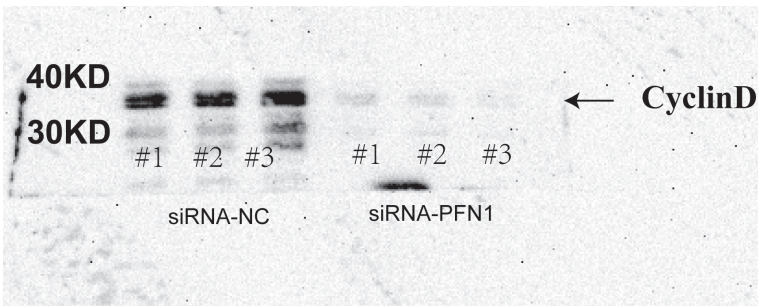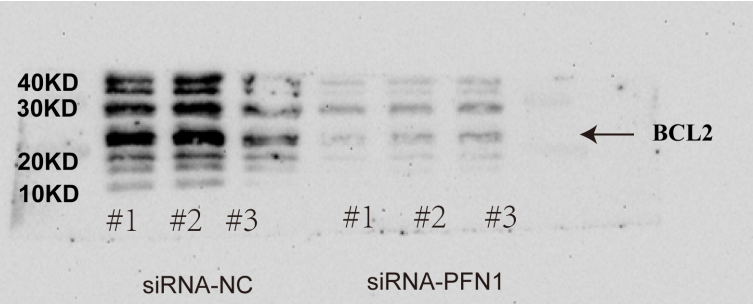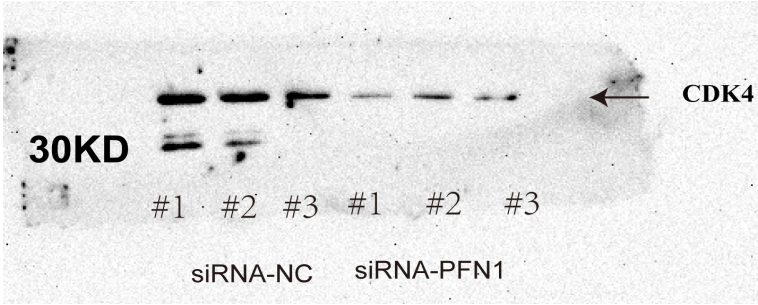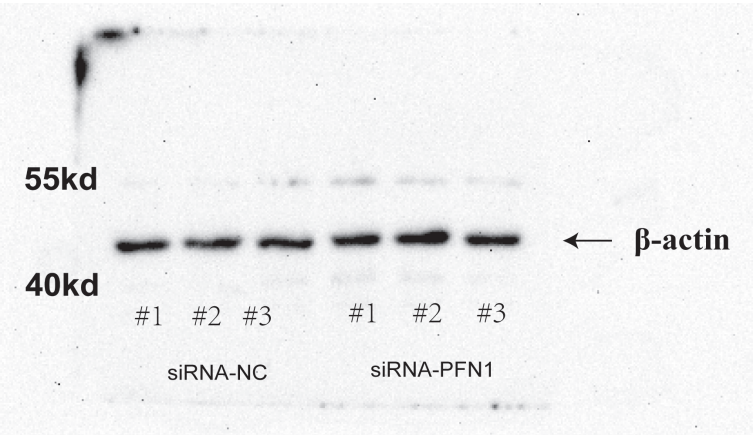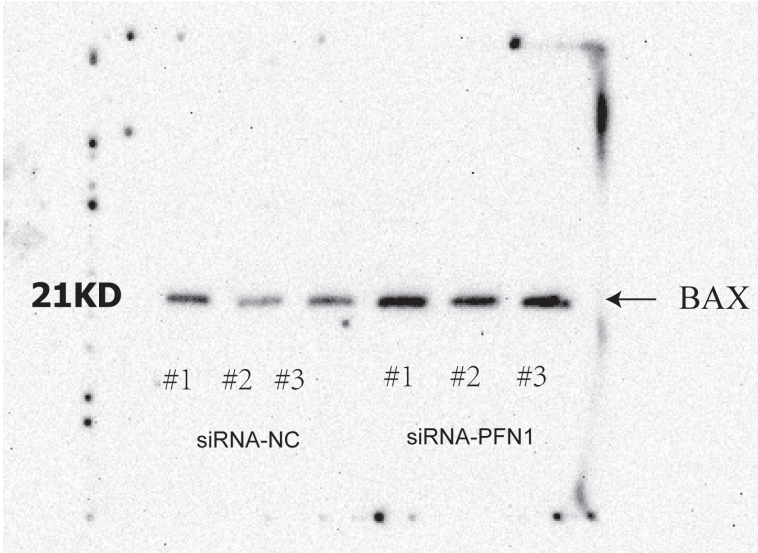

Supplement: Supplementary file 1 — Supplementary Figure S1. [file 41598_2024_65937_MOESM1_ESM.pdf]
