# Supplementary material for: Identification of a novel lactylation-related gene signature predicts the prognosis of multiple myeloma and experiment verification
Source: Sci Rep. 2024 Jul 2;14:15142. doi: 10.1038/s41598-024-65937-x (PMC11219856; doi:10.1038/s41598-024-65937-x)
Supplement: Supplementary file 4 — Supplementary Table S3. [file 41598_2024_65937_MOESM4_ESM.docx]

**Supplementary Table 3. Antibodies for Western bolt in this study**

| Antibody Name | Details |
| --- | --- |
| PFN1 | Proteintech, #3A12E8, 1:200 |
| BCL2 | CST, #3498, 1:1000 |
| BAX | CST, #2772, 1:1000 |
| CDK4 | CST, #12790, 1:1000 |
| CYCLIND1 | CST, #2922, 1:1000 |
| β-ACTIN | CST, #8457, 1:1000 |
